# Myrtenol from Lavender Essential Oil Possesses Neuroprotective Effects and Promotes Neurite Outgrowth by Potentially Targeting TrkA and IGF-1R in PC12 Cells

**DOI:** 10.3390/ijms27062615

**Published:** 2026-03-12

**Authors:** Ting Jiang, Lan Xiang, Jianhua Qi

**Affiliations:** College of Pharmaceutical Sciences, Zhejiang University, Yu Hang Tang Road 866, Hangzhou 310058, China; 22319087@zju.edu.cn

**Keywords:** neurodegeneration, natural products, oxidative stress, lavender essential oil, bioactive compounds, neuroprotection, Alzheimer’s disease, cellular mechanisms

## Abstract

Alzheimer’s disease (AD) is a prevalent chronic neurodegenerative disorder; the progression of this disease is driven by cellular determinants such as oxidative stress and dysregulated neurotrophic signaling. Lavender essential oil is traditionally used in aromatherapy for neuronal regulation and neuroprotection, suggesting its potential neuroprotective effects for chronic neurodegenerative disorders like AD. However, the key active constituents responsible for its benefits and the specific molecular pharmacological mechanisms remain unclear. In this study, we isolated myrtenol from lavender essential oil under the guidance of activity evaluation. Its neuroprotective effects were evaluated in PC12 cells via neurite outgrowth, anti-A*β*/H_2_O_2_ cytotoxicity, and antioxidant assays. Targets and pathways were explored using inhibitor experiments, cell thermal shift assay (CETSA), drug affinity responsive target stability (DARTS), and Western blot. Myrtenol significantly induced neurite outgrowth in PC12 cells and effectively mitigated cytotoxicity and oxidative stress damage induced by A*β*_25–35_ and H_2_O_2_. Mechanistic studies revealed that myrtenol’s effects are associated with the modulation of tyrosine kinase receptor A (TrkA) and insulin-like growth factor-1 receptor (IGF-1R), activating phospholipase C (PLC)/protein kinase C (PKC) and phosphoinositide 3-kinase (PI3K)/protein kinase B (AKT) signaling pathways to jointly mediate neuroprotection effects against the pathology of AD. This study demonstrates that myrtenol as a highly active component of lavender essential oil possesses NGF-like neuritogenic activity and neuroprotective effects. It provides a foundation for understanding the cellular mechanisms of myrtenol as a small-molecule lead for further investigation in neurodegeneration-related research.

## 1. Introduction

Alzheimer’s disease (AD) is the most common neurodegenerative disorder, causing cognitive decline, abnormal mental and behavioral manifestations, and loss of social functions, which constitutes a significant public health threat [1]. Although the approved drugs have demonstrated certain therapeutic effects, their high costs and limited applicability make them difficult to serve as effective universal treatment options. In recent years, aromatherapy has attracted significant attention due to its multi-target regulation properties. This therapy directly acts on the cerebral cortex, thalamus, and limbic system by inhaling or transdermal absorption of volatile small molecules in plant essential oils, regulating the levels of key neurotransmitters, thereby alleviating various symptoms in AD patients [2]. Among these, natural essential oils are not only an important component of natural product resources but also the core of aromatherapy. Studies have shown that compounds such as terpenes and phenols abundant in natural essential oils can intervene in the pathological process of AD through multiple mechanisms such as anti-A*β* deposition and inhibition of acetylcholinesterase (AChE) activity, demonstrating excellent potential for AD treatment [3,4].

Among the numerous natural essential oils with neuroprotective potential, lavender (*Lavandula angustifolia*) essential oil has become a hot topic due to its remarkable ability to counteract the core pathologies of AD. The potent antioxidative stress capacity is one of the core advantages of lavender essential oil, which effectively alleviates AD-related neuronal damage through mechanisms such as directly scavenging free radicals and activating endogenous antioxidant pathways [5]. Oxidative stress plays a critical role in the progression of AD. A growing body of evidence suggests that oxidative stress is associated with AD pathogenesis across multiple etiological hypotheses and acts as a critical nexus driving the occurrence and development of AD [6,7]. The accumulation of excessive reactive oxygen species (ROS) and lipid peroxidation products, malondialdehyde (MDA), accelerates neuronal apoptosis and synaptic dysfunction. Therefore, lead compounds with antioxidative stress activity may demonstrate greater therapeutic potential for AD treatment. Furthermore, lavender essential oil exerts synergistic neuroprotective effects through anti-A*β* toxicity and inhibition of AChE activity [8,9], and has demonstrated marked efficacy in improving learning and memory abilities in animal models [10].

AD is characterized by complex pathological features and multifactorial pathogenesis. Among these, damage to cholinergic neurons and deficiency in neurotrophic factors represent critical aspects of AD progression. For decades, nerve growth factor (NGF) therapy has been regarded as a potential breakthrough due to its targeting of the core pathology of AD. NGF promotes neuronal survival and synaptic plasticity by activating the TrkA receptor and regulating downstream signaling pathways such as PI3K/AKT and Ras/MAPK, thereby directly addressing the core pathology of AD [11]. However, its protein characteristics result in poor penetration through the blood–brain barrier (BBB), short half-life, significant individual variability in response, and side effects such as pain hypersensitivity, which severely limits the clinical application of NGF-based therapies [12]. As AD poses a major health challenge to aging societies worldwide, the development of small-molecule compounds with NGF-mimic activity not only holds potential for overcoming the limitations of protein-based drugs and offering new treatment options for AD and related diseases but also carries significant practical implications for alleviating public health burdens and promoting the research and development of neuroprotective drugs.

The exertion of NGF-mimicking functions requires specific activation of key target proteins, initiating complex intracellular signaling networks to regulate neuronal survival, differentiation, and synaptic plasticity. Classic downstream pathways include the Ras/MAPK pathway, the phosphoinositide 3-kinase/protein kinase B (PI3K/Akt) pathway, and the phospholipase C (PLC) pathway. The Ras/MAPK cascade modulates gene expression and is primarily responsible for promoting neuronal differentiation, axonal growth, and synaptic plasticity [13]. Meanwhile, the PI3K/Akt pathway serves as a crucial survival signal, exerting potent anti-apoptotic effects and supporting neuronal metabolism [14]. Activation of PLC generates second messengers that regulate intracellular calcium levels and protein kinase C (PKC) activity, influencing short-term processes such as synaptic function and neuronal excitability [15].

Given the multi-target regulatory properties of essential oils and the urgent need for effective NGF alternatives, this study aims to identify the key active components in lavender essential oil that are responsible for its neuroprotective effects. In this study, we isolated the active compound from lavender essential oil under a bioactivity-guided fractionation approach using PC12 cells. The compound responsible for the outstanding observed bioactivity was isolated and structurally elucidated as myrtenol; we then evaluated its neuroprotective effects and underlying mechanisms in PC12 cell models.

Here, we systematically evaluated the neuroprotective effects of myrtenol in PC12 cell models for the first time. The results revealed that the NGF-like neuritogenic activity of myrtenol is closely associated with potentially dual targeting TrkA and IGF-1R and activation of both the PLC/PKC and PI3K/AKT pathways.

## 2. Results

### 2.1. Effects of Myrtenol on Neurite Outgrowth of PC12 Cells

**Figure 1:**
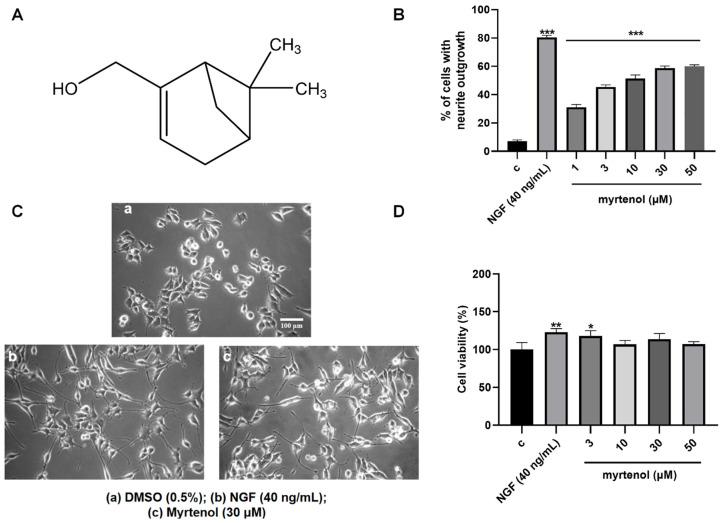
Effect of myrtenol on neurite outgrowth and cell viability in PC12 cells. (**A**) Chemical structure of myrtenol. (**B**,**C**) The percentage of neurite outgrowth and representative morphological changes in PC12 cells treated with 1, 3, 10, 30 and 50 μM myrtenol for 48 h. Scale bar = 100 μm. (**D**) Viability of PC12 cells treated with different doses of myrtenol for 24 h. Here, 0.5% DMSO was used as negative control and 40 ng/mL NGF was used as positive control. Each experiment was repeated three times. Data are presented as mean ± standard error of the mean (SEM). * *p* < 0.05, ** *p* < 0.01, and *** *p* < 0.001 indicated significant differences from the negative control groups.

The NGF-like neuritogenic activity of myrtenol was assessed with the PC12 cells. Here, 0.5% DMSO served as the negative control and 40 ng/mL NGF as the positive control. As shown in Figure 1B,C, myrtenol significantly induced neurite outgrowth in a dose-dependent manner compared to the negative control group. The percentages of PC12 cells with neurite outgrowth induced by myrtenol were 31.00% ± 1.16%, 45.33% ± 0.88%, 51.33% ± 1.45%, 58.67% ± 0.88%, and 59.00% ± 1.15% at concentrations of 1, 3, 10, 30, and 50 µM, respectively.

To assess whether myrtenol exhibits cytotoxicity toward PC12 cells within the tested concentration range, cell viability was measured using the MTT assay. After treatment with myrtenol at concentrations of 3, 10, 30, and 50 μM, the cell viability was determined to be 117.80% ± 4.07%, 106.80% ± 2.97%, 113.70% ± 4.24%, and 107.10% ± 1.79%, respectively. There was no significant reduction in cell viability compared to the control group (Figure 1D), indicating that myrtenol did not exhibit cytotoxic effects under the experimental conditions. Based on the combined results of the activity and toxicity assays, 30 μM and 50 μM myrtenol produced comparable effects. Thus, 30 μM was selected as the optimal working concentration for the subsequent experiments.

### 2.2. Myrtenol Alleviated Cell Damage Induced by Aβ and H_2_O_2_ in PC12 Cells

**Figure 2:**
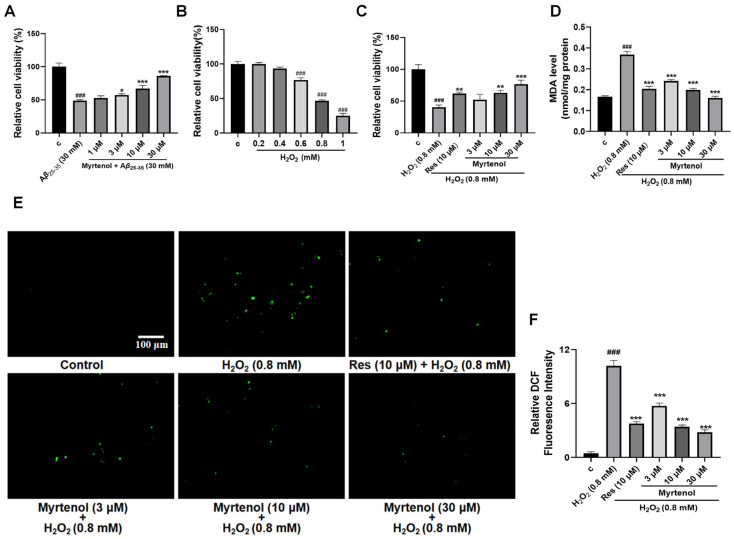
Neuroprotective activity of myrtenol against A*β*-induced cell damage and against oxidative stress. (**A**) Cell viability after induction of cell damage with A*β*_25–35_ and treatment with various concentrations of myrtenol. (**B**) PC12 cell viability change caused by different doses of H_2_O_2_. (**C**) Cell viability under oxidative stress induced by H_2_O_2_ and treated with different concentrations of myrtenol. (**D**) MDA levels were detected after treatment with different concentrations of myrtenol. (**E**,**F**) Representative fluorescence images and quantitative analysis of ROS levels in PC12 cells stained with DCFH-DA probe after treatment with different concentrations of myrtenol. Approximately 5 × 10^4^ cells were seeded into each well. Images were acquired using an Olympus IX53 inverted microscope (IX53, Olympus, Tokyo, Japan, 20× objective). Scale bar = 100 μm. Data are presented as mean ± SEM. The 0.5% DMSO treatment served as the negative control group, and resveratrol (Res, 10 μM) was used as the positive control. ^###^ *p* < 0.001 indicates significant differences compared to the negative control groups; * *p* < 0.05, ** *p* < 0.01, and *** *p* < 0.001 indicate significant differences compared to the A*β*_25–35_ or H_2_O_2_ treatment groups.

Lavender essential oil has demonstrated significant neuroprotective effects [8,9,10]. To investigate whether myrtenol, the constituent derived from lavender essential oil, also exhibits neuroprotective activity, anti-A*β* injury assay and antioxidative stress assay were used for verification. Some studies have indicated that oxidative stress promotes core pathological features of AD, including accelerating the generation and accumulation of A*β* [6,16]. In turn, the toxic effects of A*β* impair mitochondrial function, increase ROS production, and exacerbate oxidative stress. Forming a “vicious cycle” that collectively drives AD progression. In the A*β*-induced damage assay, when the cells were treated with 30 μM A*β*_25–35_, the cell viability of the model group was reduced to 48.85% ± 0.74% compared to the control group. Experimental data showed that 10 μM myrtenol treatment could increase the cell viability to 67.11% ± 6.54%, and 30 μM myrtenol treatment could increase the cell viability to 85.78% ± 0.46%, and the proportion of abnormal morphology cells was significantly reduced (Figure 2A).

In the antioxidant stress experiment, a preliminary experiment was conducted first to determine the optimal concentration of H_2_O_2_. Based on the experimental results (Figure 2B), 0.8 mM of H_2_O_2_ was used to induce cell damage. Treatment with 0.8 mM of H_2_O_2_ induced significant cell damage, reducing cell viability to 46.85% ± 1.96% compared to the control group (Figure 2C). Cells treated with 10 μM resveratrol served as the positive control. Compared with the negative control group, the cell viability of the positive group increased to 61.78% ± 1.34%. Treatment with different concentrations of myrtenol also conferred protective effects. As shown in Figure 2C, 10 μM of myrtenol produced comparable protection to resveratrol, while 30 μM of myrtenol increased cell viability to 76.59% ± 3.64% and markedly attenuated abnormal morphological changes.

### 2.3. Screening Potential Protein Targets and Signaling Pathways Related to the NGF-like Neuritogenic Effect of Myrtenol by Specific Inhibitors

**Figure 3:**
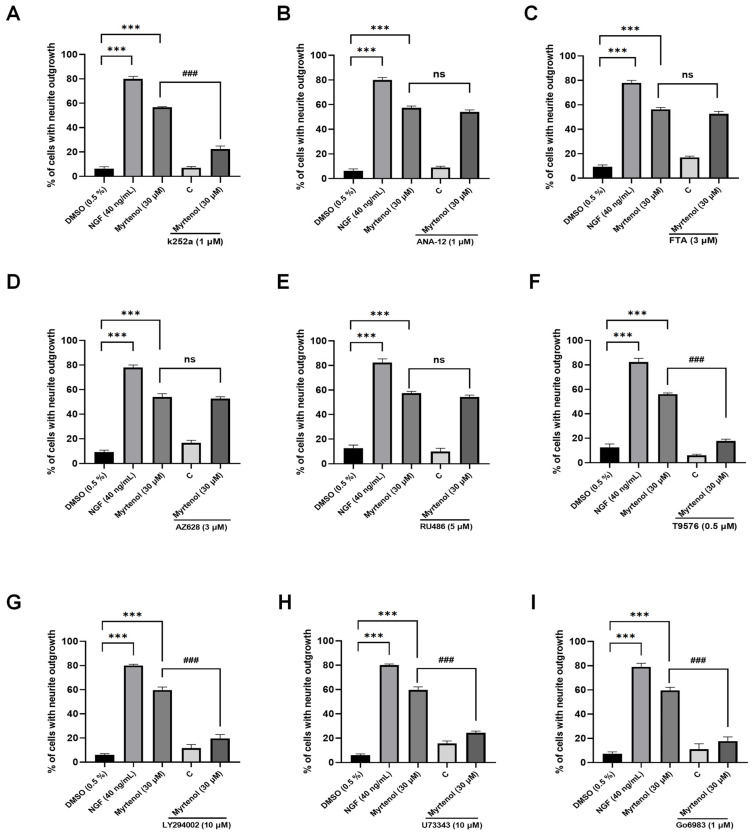
Screen for protein targets and signaling pathways related to the NGF-like neuritogenic effect of myrtenol by specific inhibitors. (**A**–**D**) Effect of classical Trks family-specific inhibitors on the NGF-like neuritogenic effect of myrtenol. Explore the effects of TrkA inhibitor (k252a), TrkB inhibitor (ANA-12), Ras inhibitor (FTA) and Raf inhibitor (AZ628) on the NGF-like neuritogenic activity induced by myrtenol. (**E**–**I**) The effects of GR inhibitor (RU486), IGF-1R inhibitor (T9576), PI3K inhibitor (LY294002), PLC inhibitor (U73343) and PKC inhibitor (Go6983) on the NGF-like neuritogenic activity induced by myrtenol were investigated. The 0.5% DMSO treatment was used as a negative control, and the 40 ng/mL NGF treatment was used as a positive control. Figure (**A**,**B**) represent the results of the same experiment, while (**C**,**D**), (**E**,**F**), and (**G**,**H**) represent the results of the same experiment, using the same control groups. Data are presented as mean ± SEM. *** *p* < 0.001 indicates significant differences compared to the negative control group; ns indicates no significant differences, and ^###^ *p* < 0.001 indicates significant differences compared with the 30 μM myrtenol treatment groups.

To elucidate the molecular mechanism underlying the NGF-like neuritogenic effects of myrtenol, we performed a preliminary screening of potential target proteins using inhibitor experiments. We first investigated whether the NGF-like neuritogenic activity involves the classic Trk family proteins involved in the nerve growth factor regulatory pathway. As shown in Figure 3A–D, the TrkA inhibitor K252a, which is involved in the classical NGF pathway, could significantly inhibit the neurite outgrowth induced by myrtenol, but its downstream ras inhibitor and raf inhibitor did not show obvious inhibitory effects. These results suggest that TrkA may be a candidate target of myrtenol, and it may play a role through other signaling pathways. Furthermore, ANA-12, the TrkB inhibitor involved in the BDNF-regulated signaling pathway, did not inhibit myrtenol-induced neurite outgrowth, indicating that the NGF-like neuritogenic activity of myrtenol is probably not mediated through TrkB.

A growing body of evidence suggests that, in addition to TrkA, cell neurite outgrowth is also regulated by the activation of multiple target proteins and involves a variety of signaling pathways [17,18,19]. We further evaluated the effect of inhibitors targeting alternative or synergistic pathways related to neurotrophic and differentiation signaling on the NGF-like neuritogenic activity of myrtenol. As shown in the results (Figure 3E), inhibition of the glucocorticoid receptor (GR) did not suppress the NGF-like neuritogenic effect of myrtenol. In contrast, the IGF-1R inhibitor T9576 significantly inhibited myrtenol-induced neurite outgrowth (Figure 3F). The downstream PI3K inhibitor LY294002 also showed a significant inhibitory effect (Figure 3G). The PLC/PKC pathway is also an important pathway in the function of TrkA. The experimental results demonstrated that both the PLC inhibitor U73343 and the PKC inhibitor Go6983 markedly suppressed the neurite outgrowth induced by myrtenol (Figure 3H,I), suggesting potential signal regulation. These findings may serve as a preliminary screening basis, indicating that the NGF-like neuritogenic activity of myrtenol may be related to the PLC/PKC and PI3K/AKT signaling pathways, and may be regulated by TrkA and IGF-1R.

### 2.4. Myrtenol Enhanced the Thermal Stability and the Stability for Pronase E of Both TrkA and IGF-1R

**Figure 4:**
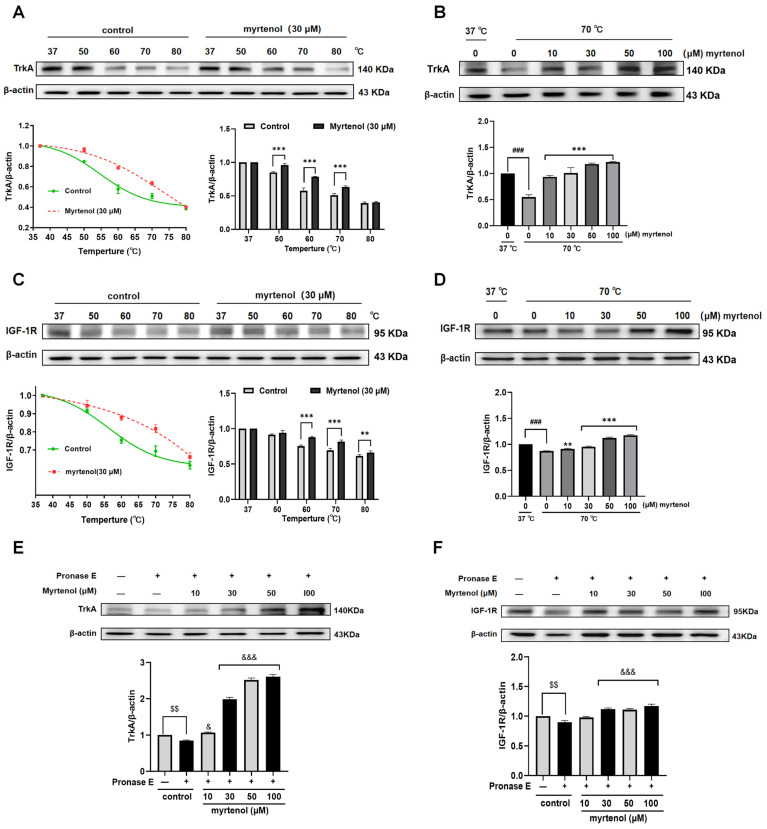
Identification of TrkA and IGF-1R as potential targets of myrtenol. (**A**) Western blot analysis and quantitative results of the interaction between myrtenol and TrkA protein using CETSA. Samples were treated with 30 µM of myrtenol and heated at temperatures ranging from 37 °C to 80 °C. (**B**) Western blot analysis and quantitative results of the binding stability between myrtenol and TrkA at varying compound concentrations. (**C**) Western blot analysis and quantitative results of the interaction between myrtenol and IGF-1R protein using CETSA. Samples were treated with 30 µM of myrtenol and heated at temperatures ranging from 37 °C to 80 °C. (**D**) Western blot analysis and quantitative results of the binding stability between myrtenol and IGF-1R at varying compound concentrations. (**E**) Western blot analysis and quantitative results of the interaction between myrtenol and TrkA protein using the DARTS assay. (**F**) Western blot analysis and quantitative results of the interaction between myrtenol and IGF-1R protein using the DARTS assay. Samples were treated with 0.5% pronase E in the presence of increasing concentrations of myrtenol, and changes in TrkA and IGF-1R protein levels were assessed by Western blot. Data are presented as mean ± SEM. ** *p* < 0.01 and *** *p* < 0.001 indicate significant differences compared with the control groups without compound treatment at 70 °C; ^###^ *p* < 0.001 indicates significant differences compared with the control groups without compound treatment at 37 °C. ^$$^ *p* < 0.001 indicates significant differences compared to the control groups without pronase E treatment; ^&^ *p* < 0.05 and ^&&&^ *p* < 0.001 indicate significant differences compared to the groups treated with pronase E alone.

We next conduct further experiments at the cellular level to verify the results of the inhibitor screening results. CETSA is a widely adopted method for target engagement studies; it evaluates drug–target interactions by detecting changes in the thermal stability of candidate proteins. PC12 cells were treated with 30 μM of myrtenol for the CETSA analysis. The results demonstrated that myrtenol enhanced the thermal stability of both TrkA and IGF-1R, leading to an increase in their thermal denaturation temperatures. As shown in Figure 4A and Figure S1A, compared to the untreated control group, myrtenol significantly improved the stability of TrkA at 50 °C, 60 °C, and 70 °C. To further confirm the binding between myrtenol and TrkA, the temperature was fixed at 70 °C while the compound concentration was varied. The results revealed that (Figure 4B and Figure S1B), in the concentration range of 0–100 μM, the stability of the interaction between myrtenol and TrkA was strengthened with the increase in the concentration, showing a dose-dependent relationship. A similar trend was observed for IGF-1R. Myrtenol treatment significantly enhanced the thermal stability of IGF-1R at 60 °C and 70 °C compared to the control group (Figure 4C and Figure S1C). When the temperature was fixed at 70 °C, increasing concentrations of myrtenol also resulted in a dose-dependent stabilization of IGF-1R (Figure 4D and Figure S1D). These findings indicate that both TrkA and IGF-1R may be the target proteins of myrtenol.

The DARTS assay is another widely used technique for small-molecule screening and target identification. We further employed the DARTS assay to validate the binding effect between myrtenol and TrkA or IGF-1R. When treated with pronase E at concentrations ranging from 0 to 2%, both TrkA and IGF-1R in the experimental group showed varying degrees of degradation (Supplementary Figure S2A,B). Treatment with 30 μM of myrtenol enhanced the resistance of TrkA and IGF-1R to pronase E-induced degradation. As shown in Figure 4E,F and Figure S1F,H, at a fixed optimal pronase E concentration of 0.5%, myrtenol at concentrations of 10, 30, 50, and 100 μM increased the resistance of both TrkA and IGF-1R against enzymatic degradation in a dose-dependent manner, and the TrkA protein was more resistant to enzymatic degradation in a dose-dependent manner. These results further support that TrkA and IGF-1R may serve as potential targets mediating the cellular effects of myrtenol.

### 2.5. The TrkA/PLC/PKC Signaling Pathway Plays an Important Role in the NGF-like Neuritogenic Activity of Myrtenol

Results from both CETSA and DARTS assays indicated that myrtenol significantly enhances the thermal stability and protease resistance of TrkA, suggesting that it may serve as the potential target for the NGF-like neuritogenic activity of myrtenol. However, inhibitor interference experiments showed that the Ras/Raf signaling pathway was not involved in this process, implying that the mechanism of myrtenol differs from the classical TrkA/Ras/Raf pathway. Previous research has reported that the phosphorylation of TrkA triggers PLC activation and triggers the cascade of the PLC/PKC pathway [15]. Combined with the experimental results presented in Figure 3, we hypothesize that the TrkA/PLC/PKC signaling pathway plays a major role in the NGF-like neuritogenic activity of myrtenol.

**Figure 5:**
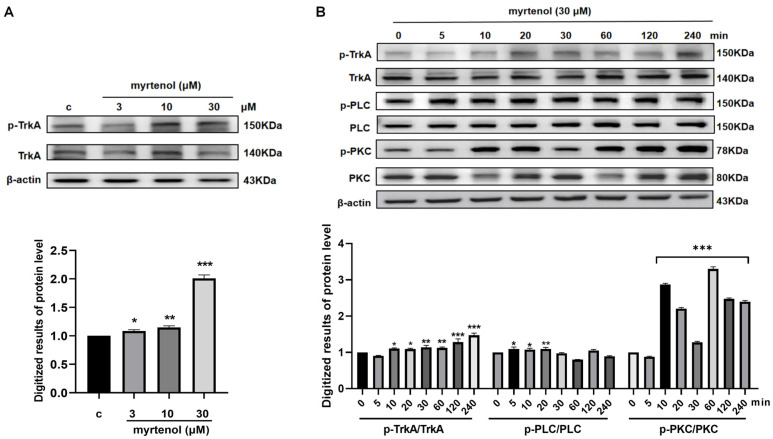
The NGF-like neuritogenic activity of myrtenol is associated with the TrkA/PLC/PKC signaling pathway. (**A**) Western blot analysis and quantitative results showing the dose-dependent induction of TrkA phosphorylation by myrtenol. (**B**) Western blot analysis and quantitative results showing the time-dependent phosphorylation of TrkA, PLC, and PKC induced by myrtenol. Data are presented as mean ± SEM. * *p* < 0.05, ** *p* < 0.01, and *** *p* < 0.001 indicate significant differences compared to the control groups.

To validate this hypothesis, we carried out a series of experiments at the cellular level. As shown in Figure 5A and Figure S3A, myrtenol treatment significantly induced TrkA phosphorylation in a dose-dependent manner. Further, cells were treated with 30 μM of myrtenol, and the time-dependent phosphorylation of TrkA, PLC, and PKC was monitored over 240 min. As illustrated in Figure 5B and Figure S3B, the phosphorylation of TrkA began to increase at 10 min and peaked at 240 min. The phosphorylation level of PLC showed a significant difference at 20 min after treatment. PKC phosphorylation was markedly enhanced starting at 10 min and remained at a high level throughout the observation period. These results demonstrate that the proteins involved in this pathway showed sensitive and durable responses to myrtenol within 240 min.

**Figure 6:**
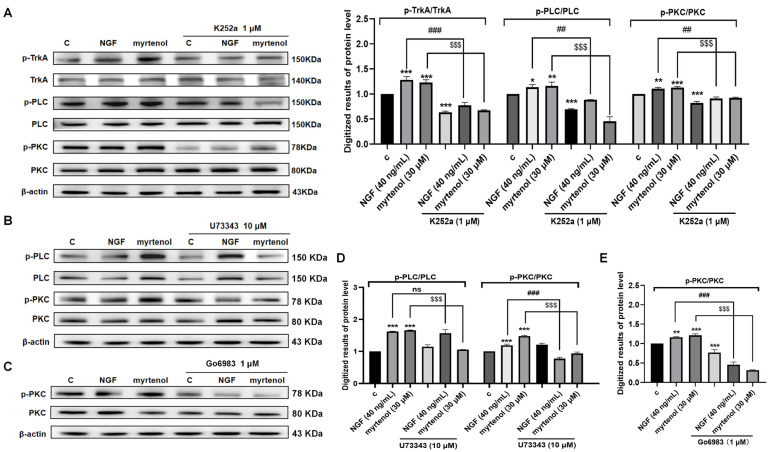
Modulation of myrtenol-induced TrkA/PLC/PKC signaling in PC12 cells by specific inhibitors. (**A**) Western blot analysis and quantitative results of phosphorylated TrkA, PLC, and PKC following treatment with the TrkA inhibitor. (**B**) Western blot analysis of phosphorylated PLC and PKC after treatment with the PLC inhibitor. (**C**) Western blot analysis of phosphorylated PKC after treatment with the PKC inhibitor. (**D**) Quantitative results of the Western blot analysis shown in (**B**). (**E**) Quantitative results of the Western blot analysis shown in (**C**). Data are presented as mean ± SEM. * *p* < 0.05, ** *p* < 0.01, and *** *p* < 0.001 indicate significant differences compared to the control groups; ns, ^##^ *p* < 0.01, and ^###^ *p* < 0.001 indicate no significant differences or significant differences compared to the 40 ng/mL NGF treatment groups; ^$$$^ *p* < 0.001 indicates significant differences compared with the 30 μM myrtenol treatment groups.

Subsequently, specific inhibitors of TrkA, PLC, and PKC were used to investigate their effects on the phosphorylation of downstream proteins. The results (Figure 6A and Figure S4A) showed that treatment with the TrkA inhibitor K252a almost completely suppressed myrtenol-induced phosphorylation of TrkA, PLC, and PKC. The PLC inhibitor U73343 significantly reduced the phosphorylation levels of both PLC and PKC induced by myrtenol (Figure 6B,D and Figure S4B), while the PKC inhibitor Go6983 also effectively inhibited the increase in PKC phosphorylation (Figure 6C,E, and Figure S4C). These findings indicate that myrtenol triggers the cascade activation of the PLC/PKC signaling pathway through TrkA.

### 2.6. The IGF-1R/PI3K/AKT Signaling Pathway Is Also Involved in the NGF-like Neuritogenic Activity of Myrtenol

**Figure 7:**
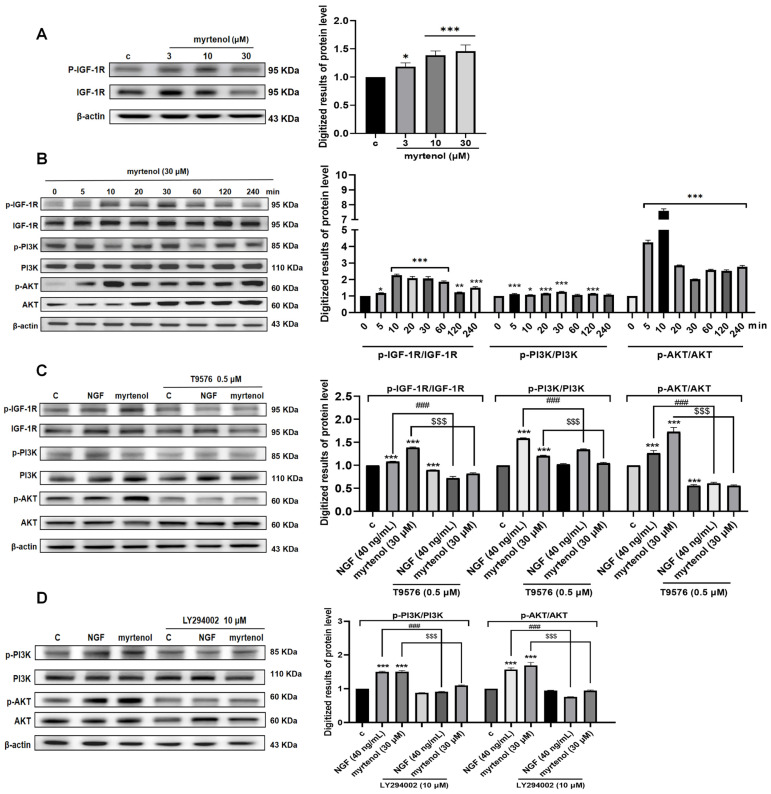
The NGF-like neuritogenic activity of myrtenol is associated with the IGF-1R/PI3K/AKT signaling pathway. (**A**) Western blot analysis and quantitative results showing the dose-dependent induction of IGF-1R phosphorylation by myrtenol. (**B**) Western blot analysis and quantitative results showing the time-dependent phosphorylation of IGF-1R, PI3K, and AKT induced by myrtenol. (**C**) Western blot analysis and quantitative results of phosphorylated IGF-1R, PI3K, and AKT after treatment with the IGF-1R inhibitor. (**D**) Western blot analysis and quantitative results of phosphorylated PI3K and AKT after treatment with the PI3K inhibitor. Data are presented as mean ± SEM. * *p* < 0.05, ** *p* < 0.01, and *** *p* < 0.001 indicate significant differences compared to the control groups; ^###^ *p* < 0.001 indicates significant differences compared to the 40 ng/mL NGF treatment groups; ^$$$^ *p* < 0.001 indicates significant differences compared with the 30 μM myrtenol treatment groups.

In addition to TrkA, we also identified IGF-1R as a potential target of myrtenol in its NGF-like neuritogenic effects. The PI3K/AKT signaling pathway represents the most classical and core signal transduction arm of IGF-1R downstream. To systematically validate the role of this pathway, we carried out a series of experiments at the cellular level. We first examined the effect of myrtenol on IGF-1R phosphorylation. The results (Figure 7A and Figure S5A) demonstrated that myrtenol induced IGF-1R phosphorylation in a dose-dependent manner. Cells treated with 30 μM of myrtenol were then used to investigate the time-dependent phosphorylation of IGF-1R, PI3K, and AKT over 240 min. As shown in Figure 7B and Figure S5B, the phosphorylation of IGF-1R began to increase at 5 min and a significant difference was observed at 10 min. Phosphorylation of PI3K peaked as early as 5 min after treatment. The phosphorylation response of AKT was even more sensitive, as it began to increase significantly at 5 min, it reached its peak at 10 min, and it remained at a high level thereafter.

Subsequently, specific inhibitors of IGF-1R, PI3K, and AKT were used to explore their effects on the phosphorylation of downstream proteins. As shown in Figure 7C and Figure S5C, treatment with IGF-1R inhibitor T9576 almost completely inhibited myrtenol-induced phosphorylation of IGF-1R and downstream PI3K and AKT. Similarly, the PI3K inhibitor LY294002 significantly reduced the phosphorylation of PI3K and AKT induced by myrtenol (Figure 7D and Figure S5D). These results suggest that myrtenol activates IGF-1R to trigger the downstream PI3K/AKT signaling response, and that the IGF-1R/PI3K/AKT signaling pathway plays an important role in the NGF-like neuritogenic activity of myrtenol.

## 3. Discussion

The pathological process of AD involves multiple mechanisms. At present, most of the drugs used in clinical practice target a single target and have limited efficacy. Therefore, exploring small-molecule compounds derived from natural products with multi-target modulatory capabilities has become an important strategy for discovering novel therapeutic candidates.

Previous studies have shown that lavender essential oil exhibits sedative, anti-anxiety and memory-enhancing effects, which are closely related to neuroplasticity and neuroprotection [8,9,10]. However, its active components and mechanisms of action are still unclear. Our team has long been dedicated to discovering candidate molecules with anti-AD potential from natural products. We have previously identified *β*-cyclocitral from *Lavandula angustifolia Mill* as a small molecule with both NGF-like neuritogenic and NGF-enhancer activities [20]. Both myrtenol and *β*-cyclocitral demonstrate NGF-like neuritogenic effects, but they have significant differences in structure and function. *β*-cyclocitral is an aldehyde monoterpene, and mechanistic studies indicate that it promotes neurite outgrowth by targeting the IGF-1R/GR pathway. In contrast, myrtenol is a terpenoid alcohol with greater lipophilicity, and its effects are more biased toward signal transduction mediated by the TrkA receptor and IGF-1R.

During the process of target screening and validation, we employed specific inhibitors for preliminary assessment. Inhibitor-based assays serve as a critical step bridging phenotypic screening and target identification in small-molecule drug discovery, laying a foundation for more in-depth mechanistic studies. Under the existing experimental conditions in our laboratory, this strategy balanced efficiency and accuracy, laying the foundation for the subsequent verification of label-free methods such as CETSA and DARTS. We initially focused on the classical “NGF–Trk” pathway and selected corresponding inhibitors to evaluate whether myrtenol acts through the Trk family. As shown in Figure 3, TrkA was implicated in the NGF-like neuritogenic activity of myrtenol, whereas TrkB showed little correlation. Neurite outgrowth is also regulated by the activation of multiple target proteins and involves crosstalk among several signaling pathways. For example, brain-derived neurotrophic factor (BDNF) promotes neuronal differentiation through TrkB activation, involving the activation of downstream pathways such as Ras/Raf and PLC/PKC [17]; the phosphorylation of IGF-1R and INSR can trigger cascade reactions of downstream signaling pathways [18]; and the GR is also known to modulate neuronal structure and plasticity [19]. Therefore, we further evaluated whether these neurotrophic/differentiation-related alternative or synergistic pathway inhibitors could suppress the NGF-like neuritogenic effects of myrtenol.

It is noteworthy that the activation of TrkA by myrtenol did not trigger the classical Ras/Raf signaling cascade but preferentially induced the PLC/PKC signaling cascade. The PLC/PKC pathway is closely associated with synaptic plasticity, neurotransmitter release, and learning and memory, playing a crucial role in improving cognitive function [21]. This atypical activation pattern may be related to its specific binding conformation or allosteric regulatory mechanism, warranting further investigation. On the other hand, a previous study indicated that insulin/IGF-1 signaling resistance is commonly observed in the brains of AD patients [22]. Activating IGF-1R can directly counteract cerebral insulin resistance, restore normal function of the downstream PI3K/AKT pathway, and thereby improve neuronal energy metabolism, enhance synaptic plasticity, and suppress tau protein pathology. The efficient activation of the IGF-1R/PI3K/AKT pathway by myrtenol is closely linked to its significant anti-apoptotic and pro-survival effects. The PI3K/AKT pathway is not only a classical survival and anti-apoptotic signaling axis but is also deeply implicated in impaired insulin signaling and abnormal neuronal metabolism in AD pathogenesis [23]. The activation has direct implications for delaying neurodegenerative diseases. The activation of both TrkA and IGF-1R may not only enhance neurite outgrowth but also provide more comprehensive neuroprotection within the multifactorial pathological environment of AD.

Furthermore, in the oxidative stress assays, myrtenol demonstrated significant antioxidant activity. Oxidative stress is considered to be the core event in the progression of AD, where excessive reactive oxygen species and lipid peroxidation will accelerate neuronal damage. The ability of myrtenol to reduce oxidative stress and improve cell survival suggests that its neuroprotective effects may not only rely on the activation of TrkA- and IGF-1R-mediated downstream signaling pathways but may also be complemented by its antioxidant properties. This function synergizes with its neurotrophic effects, together constituting its potential for multi-dimensional intervention in AD pathology.

Myrtenol is a natural monoterpene compound and an important intermediate in chemical synthesis. Although its anti-inflammatory and anti-fungal properties have been reported, its potential neuroprotective effects and underlying mechanisms in AD models remain poorly explored [24]. Huang et al. demonstrated that intraperitoneal administration of myrtenol at 30 mg/kg/day produced significant neuroprotective effects in a rat cerebral ischemia model, with no observed toxicity even at doses up to 1.3 g/kg [25]. Additionally, its lipophilic nature (log P ≈ 2.8) and measurable aqueous solubility (426.9 mg/L at 25 °C) suggest physicochemical characteristics potentially favorable for absorption [26]. However, systematic pharmacokinetic studies are needed to confirm the in vivo brain concentrations of myrtenol. Additionally, given its volatile and lipophilic nature, myrtenol may be amenable to further exploration in delivery models relevant to aromatherapy; however, such applications remain speculative at this stage and would require validation in appropriate in vivo systems.

In the in vitro model studies of AD, a common method for modeling is to treat cells with different fragments or full-length A*β* peptides. In this study, the A*β*_25–35_ fragment was used to induce damage in PC12 cells, establishing an in vitro activity evaluation model for AD therapeutic drugs. A*β*_25–35_ is the core toxic fragment of A*β*_1–42_, retaining the physical and biological properties of the full-length A*β*, and having a conformation and neurotoxicity feature similar to that of the full-length peptide [27]. Research has found that A*β*_25–35_ can spontaneously aggregate into *β*-folded structures and form fibers in vitro, closely resembling the pathological deposits in the brains of AD patients [28]. Compared to full-length A*β* peptides, A*β*_25–35_ induces a faster and more reproducible modeling effect, and it can induce obvious neurotoxicity at lower concentrations in a shorter timeframe, making it particularly suitable for pharmacodynamic evaluation at the cellular level.

Lavender essential oil exhibits notable neuroprotective activities. Traditional research usually focused on the major constituents, but myrtenol has no advantage in content. We propose that the overall bioactivity of lavender essential oil may be driven by highly potent minor constituents rather than the major components, or may arise from combinatory effects of multiple compounds acting through a synergistic network involving multi-component, multi-target, and multi-pathway mechanisms. It is insufficient for us to interpret only based on a single compound. Future studies should combine the methods of component interaction analysis, network pharmacology, and systems biology approaches to comprehensively elucidate the mechanism of action of lavender essential oil. As research progresses, we are increasingly convinced that lavender essential oil may harbor additional critical active molecules with promising neuroprotective properties. The discovery of such compounds offers an attractive new strategy to overcome the current limitations of NGF-based therapies and also provides an important foundation for the discovery of small-molecule anti-AD lead compounds from natural sources.

In this study, we selected PC12 cells as the experimental model. PC12 cells are a rat adrenal pheochromocytoma-derived cell line. They can differentiate into neurites under the stimulation of NGF, making them a classical and widely used model for studying neurite outgrowth in neuropharmacological research [29]. However, as a tumor-derived cell line, their metabolic state differs from that of normal neurons, and they lack the key AD pathological features such as A*β* plaques and neurofibrillary tangles [30]. Although we induced partial pathological phenotypes using A*β*_25–35_ and H_2_O_2_, this model still does not fully recapitulate the complex neurodegenerative environment of AD. Importantly, it does not address other critical aspects of the disease, including tau pathology, synaptic transmission, neuronal network activity, or behavioral outcomes. Therefore, in the future, we will consider using more comprehensive models such as primary neurons, human-derived systems, and in vivo AD models to determine the translational relevance of myrtenol.

The target identification approaches employed in our study also have inherent limitations. We used inhibitors for pathway dissection; however, pharmacological inhibitors such as K252a and LY294002 have inherent specificity limitations and cannot conclusively establish direct receptor binding or precise signaling hierarchies [31,32]. Therefore, these findings should be considered as preliminary screening evidence guiding subsequent validation. Subsequently, we conducted CETSA and DARTS experiments for target validation. While CETSA and DARTS are widely used in natural product research to assess cellular target engagement, these techniques do not provide structural confirmation of direct binding [33,34]. In the future, we will consider using techniques such as small interfering RNA (siRNA), surface plasmon resonance (SPR), or molecular docking to provide more conclusive evidence for the mode of interaction between myrtenol and TrkA/IGF-1R.

We propose that myrtenol is a promising naturally derived NGF-like neuritogenic compound. Its potential dual-target mechanism and pleiotropic features not only offer a novel strategic approach for the prevention and treatment of AD but also provide an experimental basis for the modernization and scientific validation of aromatherapy. Although this study has yielded positive results at the cellular level, further validation of its efficacy and safety in more complex systems is still required. Future research should focus on the structural optimization, formulation development, and in vivo pharmacodynamic evaluation of myrtenol to facilitate its translation into clinical applications.

## 4. Materials and Methods

### 4.1. Materials and Reagents

The lavender essential oil used in the experiment was purchased from Thursday Plantation overseas flagship store (Product ID 538290768594). Chromatographic column silica gel powder and TLC silica gel plate were purchased from Yantai Research Institute of Chemical Industry, Yantai, China. Analytical-grade n-hexane and dichloromethane were purchased from Sinopharm Chemical Reagent Co., Ltd., Shanghai, China. Chromatographic-grade methanol was purchased from Tedia Company, Fairfield, OH, USA. The inhibitors and antibodies used in the experiments are listed in Supplementary Table S1. All inhibitors were dissolved in DMSO and were stored as stock solutions at −30 °C.

### 4.2. Cell Lines and Culture Conditions

PC12 cells were purchased from the Cell Bank of the Chinese Academy of Sciences, Shanghai, China. Dulbecco’s Modified Eagle Medium (DMEM) was purchased from CellMax Cell Technology Co., Ltd., Beijing, China. Horse serum (HS) and fetal bovine serum (FBS) were purchased from Gibco, Herndon, VA, USA, and Penicillin–Streptomycin solution was purchased from Solarbio Science & Technology Co., Ltd., Beijing, China. DMSO and NGF were purchased from Sigma-Aldrich Co., Ltd., Boston, MA, USA.

The CM medium used in the experiments was formulated as follows: DMEM supplemented with 10% HS, 7.5% FBS, and 1% Penicillin–Streptomycin solution. PBS was prepared as follows: 4 g of NaCl, 0.1 g of KCl, 0.575 g of Na_2_HPO_4_, and 0.01 g of KH_2_PO_4_ were dissolved in 500 mL of miliQ water, sterilized by autoclaving, and stored in a refrigerator at 4 °C after cooling.

### 4.3. Isolation and Structure Identification of Myrtenol

Lavender essential oil (1 g) was fractionated using silica open-column chromatography with an n-hexane/dichloromethane solvent system (10:0, 9:1, 8:2, 7:3, 0:10 (*v*/*v*)). Fractions were combined into three crude fractions based on TLC analysis. The samples were tested for the NGF-like neuritogenic activity in PC12 cells, and the fraction (296 mg) with the best activity was further purified by HPLC (Cosmosil 5C18-MS-II column [10 × 250 mm]) (Nacalai Tesque, Kyoto, Japan). The solvent system used was a mixture of MeCN (A) and H_2_O (B) in gradient mode: 45% to 80% A for 20 min followed by 80% to 100% A for 5 min, then 100% A for 20 min; flow rate: 3 mL/min; detection wavelength: 210 nm. The structure of the molecule (2.1 mg, t_R_ = 17.18 min) with significant NGF-like neuritogenic activity was elucidated by ^1^H NMR and HR ESI-MS. The ^1^H NMR (500 MHz, CDCl_3_) data are as follows: *δ* (ppm) = 5.47 (1H, s), 3.99–3.98 (2H, m), 2.44–2.10 (5H, m), 1.45 (1H, br.s), 1.29 (3H, s), 1.17 (1H, d, J = 8.6), 0.83 (3H, s). HR ESI-TOF-MS *m*/*z* 153.1277, calculated for C_10_H_16_O [M+H]^+^ 153.1274. It was identified as myrtenol compared with the reference [35], and its structure is shown in Figure 1A. The ^1^H NMR spectrum of myrtenol is shown in Supplementary Figure S6.

### 4.4. Neurite Outgrowth Activity Assay in PC12 Cells

In this study, the PC12 cells were selected to evaluate the NGF-mimic activity of the compounds. After resuscitation, PC12 cells were seeded into 10 cm culture dishes for growth. The culture medium was replaced every 24 h. When cell density reached approximately 80%, the cells were passaged. For subculture, cells were plated into 24-well plates at a density of 5 × 10^4^ cells per well. After 24 h, the liquid in each well was replaced by DMEM containing 0.5% DMSO, NGF (40 ng/mL), or myrtenol (1, 3, 10, 30, 50 µM) and incubated in an incubator for 48 h. A cell was scored as positive for neurite outgrowth if it met the following criteria: (i) the cell possessed at least one neurite with a length equal to or greater than twice the diameter of the cell body, and (ii) the neurite exhibited a clear growth cone at its tip and did not result from cellular debris or fragmentation. For each well, three randomly selected fields were imaged under an inverted microscope (CKX41, Olympus, Tokyo, Japan, 20× objective), with 150–200 cells counted per field. The rate of positive cells was calculated as follows: (the number of positive cells/total number of cells counted) × 100%.

### 4.5. Cell Viability Assay Using MTT Method

The methods for cell inoculation and sample addition are as described in Section 4.4. After treatment, 500 µL of DMEM containing 0.2 mg/mL 3-(4,5-dimethylthiazol-2)-2,5-diphenyltetrazolium bromide (MTT) (Richu BioScience Co., Ltd., Shanghai, China) was added to each well and incubated for another 2 h. Subsequently, the supernatant was carefully aspirated, and 200 µL of DMSO was added to each well. The mixture was shaken gently to completely dissolve the purple formazan crystals. Each sample was tested in triplicate. Finally, the absorbance value of each well was measured at a wavelength of 570 nm using a plate reader (BioTek Synergy H1, Agilent, Winooski, VT, USA), and the cell viability was calculated based on the absorbance values.

### 4.6. Assessment of Neuroprotective Activity in PC12 Cells

For the A*β*-induced cytotoxicity assay, A*β*_23–35_ was first dissolved in hexafluoroisopropanol (HFIP) and allowed to stand overnight at room temperature for solvent evaporation. The resulting peptide was then reconstituted in DMSO to obtain a stock solution of monomeric A*β*_23–35_. This was subsequently diluted with DMEM and incubated at 37 °C for 3 days to form A*β*_23–35_ oligomers for subsequent experiments.

Next, approximately 1 × 10^4^ PC12 cells were seeded into each well of a 96-well plate and cultured for 24 h. Then, 100 µL of DMEM containing 0.5% DMSO or myrtenol (1, 3, 10, 30 µM) was added to each well. After 30 min of incubation, the cells were treated with 30 µM of A*β*_25–35_ and further incubated for 48 h, and cell viability was assessed using the MTT assay method.

To test cell viability against H_2_O_2_-induced oxidative stress, approximately 5 × 10^4^ PC12 cells were seeded into each well of a 24-well plate and cultured for 24 h. The cells were then treated with 0.5% DMSO, 10 µM of resveratrol (Shanghai Yuanye Bio-Technology Co., Ltd., Shanghai, China) or myrtenol (3, 10, 30 µM), and incubated for another 24 h. After removal of the medium, the cells were exposed to DMEM containing 0.8 mM of H_2_O_2_ for 1 h. Cell viability was subsequently measured using the MTT assay method, and each sample was tested in triplicate.

### 4.7. The Measurement of ROS and MDA Levels

The ROS levels in PC12 cells were detected by the 2,7-dichlorodihydrofluorescein diacetate (DCFH-DA) fluorescent probe (Beyotime Biotechnology Inc., Shanghai, China). Approximately 5 × 10^4^ cells were seeded into each well of a 24-well plate and cultured for 24 h. The cells were then treated with 0.5% DMSO, resveratrol (10 µM) or myrtenol (3, 10, 30 µM), and incubated for another 24 h; each sample was tested in triplicate. After removal of the medium, the cells were exposed to DMEM containing 0.8 mM of H_2_O_2_ for 1 h. The supernatant was then aspirated, and 500 µL of DMEM containing 10 µM of DCFH-DA was added to each well. After incubation in the dark for 30 min, the supernatant was removed, and the cells were gently washed with PBS. Then, 1 mL of PBS was added to each well. Images were acquired using a fluorescence microscope (IX53, Olympus, Tokyo, Japan, 20× objective), and the relative ROS levels were evaluated based on fluorescence intensity.

For the determination of MDA levels, about 2 × 10^6^ PC12 cells were seeded into each 6 cm culture dish and cultured for 24 h. Then, the cells were treated with 0.5% DMSO, resveratrol (10 µM), or myrtenol (3, 10, 30 µM) for another 24 h before exposure to 0.8 mM of H_2_O_2_ for 1 h. Each sample was tested in triplicate. Subsequently, cellular proteins were collected and quantified. The MDA content was determined using the MDA assay kit (Nanjing Jiancheng BioEngineering Institute, Nanjing, China).

### 4.8. Western Blot Analysis

To determine the expression level of the target protein at the cellular level, it is first necessary to prepare protein samples. The methods for cell inoculation and sample addition are as described in Section 4.4. After discarding the supernatant, the cells were washed three times with PBS. Then, 150 µL of radio immunoprecipitation assay (RIPA) lysis buffer containing 1% protease inhibitor and 1% phosphatase inhibitor was added. The cells were scraped off using a cell scraper and transferred to a 1.5 mL centrifuge tube. The lysate was kept on ice for 20 min, followed by centrifugation at 12,000 rpm and 4 °C for 20 min. The resulting supernatant was then transferred to a new centrifuge tube, which was the sample protein stock solution.

The concentration of the protein stock solution was determined using the BCA kit (CoWin Biotech Co., Ltd., Taizhou, China). The standard protein bovine serum albumin (BSA) solution was diluted into 6 concentrations to determine the standard curve. The original protein stock solution was diluted 10 times, and 25 µL of each diluted BSA standard solution or protein sample was added to each well of a 96-well plate. Then, 200 µL of the prepared working reagent was added to each well, followed by incubation at 37 °C for 30 min. The absorbance at 562 nm was measured, with each sample tested in triplicate. Protein concentration was calculated based on the standard curve. Then, sodium dodecyl sulfate polyacrylamide gel electrophoresis (SDS-PAGE) loading buffer (Beijing CoWin Biotech Company, Beijing, China) was added to each sample. Heating occurred at 100 °C for 10 min to induce protein denaturation.

For Western blot analysis, equal amounts of protein samples (20 µg) were separated by SDS-PAGE and transferred to polyvinylidene fluoride (PVDF) membrane. The membrane was blocked with 5% skim milk or BSA at room temperature for 2 h, followed by three washes with TBS with Tween-20 (TBST) (Servicebio Technology Co., Ltd., Wuhan, China). It was then incubated with diluted primary antibody (1:1000, *v*/*v*) at 4 °C overnight. The next day, after washing the PVDF membrane three times with TBST solution, the membrane was incubated with the HRP-conjugated secondary antibody at room temperature for 1 h. After washing again, bands were visualized and detected using the SuperPico ECL chemiluminescence detection kit (Vazyme Biotechnology Company, Nanjing, China). The gray values of the protein bands were quantified by ImageJ software (Version 1.42q, National Institutes of Health, Rockville, MD, USA) and normalized using *β*-actin as an internal reference. All Western blot experiments were performed with three independent biological replicates.

### 4.9. Cell Thermal Shift Assay (CETSA)

Firstly, 2 × 10^6^ cells were added into each 6 cm dish and incubated for 24 h. Control groups were treated with 0.5% DMSO; other dishes were treated with 30 µM of myrtenol and incubated for another 24 h. Cellular proteins were collected and their concentrations were determined and then quantified at 2 µg/µL. The protein samples from both the control and compound-treated groups were equally divided into five different temperature treatment groups, respectively. The samples were heated at 37 °C, 50 °C, 60 °C, 70 °C, and 80 °C for 5 min to induce varying degrees of protein denaturation. After heating, the samples were centrifuged at 12,000 rpm for 15 min. The supernatant was collected, mixed with SDS-PAGE loading buffer, and denatured by heating at 100 °C for 10 min. The thermal stability of the target protein in both control and compound-treated groups was assessed by Western blot. The CETSA experiments were repeated three independent times. Protein band intensities were quantified using ImageJ software to compare the differences in thermostability before and after compound treatment.

### 4.10. Drug Affinity Responsive Target Stability (DARTS)

The protein of PC12 cells was collected and quantified at 2 µg/µL. The optimal enzyme concentration to degrade the target protein was first explored. The protein solution in the control groups was treated with 0.5% DMSO, and the sample test groups were treated with 30 μM of myrtenol, followed by incubation at room temperature for 3 h. Then, each group of protein solutions was divided into six groups equally. Then, pronase E (MedChemExpress, Shanghai, China) was added to these groups, and final concentrations of 0, 0.02%, 0.05%, 0.2%, 0.5%, and 2% were achieved (pronase E: protein = *w*/*w*). After incubation at room temperature for 25 min, the SDS-PAGE loading buffer was added. Then, it was denatured by heating at 100 °C for 10 min. The changes in the target protein were analyzed by Western blot.

After establishing the optimal enzyme concentration, the resistance of different concentrations of samples to the enzyme degradation was explored. PC12 cell proteins were collected and quantified at 2 µg/µL. Then, the protein solution was divided into five groups, treated with 0, 10, 30, 50, and 100 μM of myrtenol and incubated at room temperature for 3 h. After that, pronase E was added at the predetermined optimal concentration. After 25 min of incubation at room temperature, the SDS-PAGE loading buffer was added, and then samples were denatured at 100 °C for 10 min. The changes in the target protein were detected by Western blot. DARTS experiments were performed with three independent biological replicates, and protein band intensities were quantified using ImageJ software.

### 4.11. Statistical Analysis

Statistical analyses were performed using GraphPad Prism 8.0 (GraphPad Software, San Diego, CA, USA). Prior to parametric analysis, data distribution was assessed using the Shapiro–Wilk normality test. An unpaired *t*-test was used for comparisons between two groups, and one-way ANOVA followed by Dunnett’s multiple comparisons test was applied to make comparisons among multiple groups. All experiments were performed with independent biological replicates (*n* ≥ 3). Data are presented as mean ± SEM, and statistical significance was defined as * *p* < 0.05.

## 5. Conclusions

**Figure 8:**
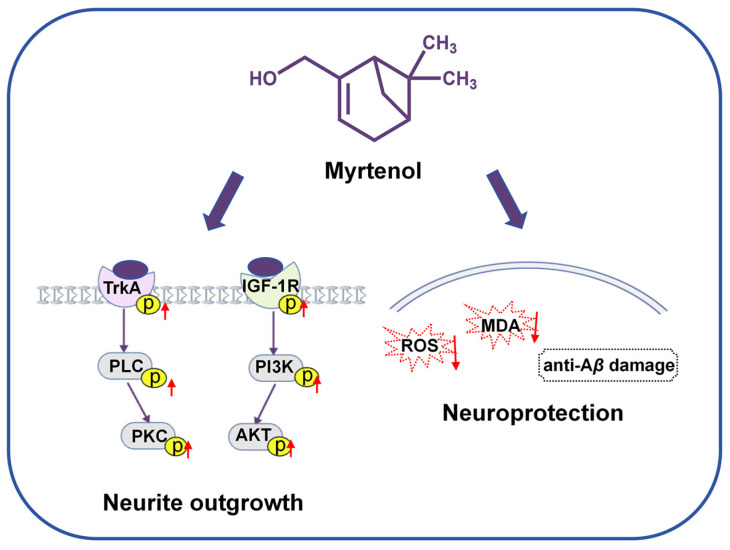
The potential mechanism of myrtenol. Myrtenol may exert the NGF-like neuritogenic effect potentially through dual targeting of TrkA/PLC/PKC and IGF-1R/PI3K/AKT signaling pathways. It also has antioxidant stress and anti-A*β* damage neuroprotective activities. The upward red arrow indicates an increase in the protein phosphorylation level. The downward red arrow represents a reduction in the ROS and MDA levels.

This study systematically demonstrates that myrtenol, a compound present in lavender essential oil, significantly promotes neurite outgrowth in PC12 cells by targeting TrkA and IGF-1R as potential target proteins, thereby activating both the PLC/PKC and PI3K/AKT signaling pathways. It also exhibits notable antioxidant and anti-A*β* toxic effects (Figure 8). This research suggests that myrtenol may serve as a natural-source candidate for AD therapy, which warrants further in-depth mechanistic investigation.

## Data Availability

The original contributions presented in this study are included in the article/Supplementary Materials. Further inquiries can be directed to the corresponding authors.

## Supplementary Materials

The following supporting information can be downloaded at: https://www.mdpi.com/article/10.3390/ijms27062615/s1.

## Abbreviations

The following abbreviations are used in this manuscript:

AD	Alzheimer’s disease
AChE	Acetylcholinesterase
A*β*	*β*-amyloid protein
AKT	Protein kinase B
BNDF	Brain-Derived Neurotrophic Factor
DCFH-DA	2,7-dichlorodihydrofluorescein diacetate
DMSO	Dimethyl sulfoxide
HPLC	High-performance liquid chromatography
IGF-1R	Insulin-like growth factor-1 receptor
MTT	3-(4,5-dimethylthiazol-2)-2,5-diphenyltetrazolium bromide
MDA	Malondialdehyde
NGF	Nerve growth factor
PI3K	Phosphoinositide 3-kinase
PBS	Phosphate-buffered solution
RIPA	Radio immunoprecipitation assay
ROS	Reactive oxygen species
SDS-PAGE	Sodium dodecyl sulfate polyacrylamide gel electrophoresis
TLC	Thin layer chromatography
TrkA	Tyrosine kinase receptor A
TNC buffer	Tris-NaCl-Tween Buffer solution
