# Supplementary material for: Myrtenol from Lavender Essential Oil Possesses Neuroprotective Effects and Promotes Neurite Outgrowth by Potentially Targeting TrkA and IGF-1R in PC12 Cells
Source: Int J Mol Sci. 2026 Mar 12;27(6):2615. doi: 10.3390/ijms27062615 (PMC13026966; doi:10.3390/ijms27062615)
Supplement: Supplementary file 1 [file ijms-27-02615-s001.zip › ijms-4145076-supplementary.pdf]

Supplementary information

Myrtenol from Lavender Essential Oil Possesses Neuroprotective Effects and Promotes Neurite Outgrowth by Potentially Targeting TrkA and IGF-1R in PC12 Cells

Ting Jiang [1], Lan Xiang [1,*] and Jianhua Qi [1,*]

[1] College of Pharmaceutical Sciences, Zhejiang University, Yu Hang Tang Road 866, Hangzhou 310058, China; 22319087@zju.edu.cn (T.J.)

* Correspondence: lxiang@zju.edu.cn (L.X.); qijianhua@zju.edu.cn (J.Q.); Tel.: +86-0571-8820-8627 (J.Q.)

Supplementary Table

Supplementary Table S1. Inhibitors and antibodies used in the present study.

Materials and Reagents	Company
TrkA inhibitor (K252a)<br>PI3K inhibitor (LY294002)<br>GR inhibitor (RU486)	Sigma-Aldrich Co, Boston, MA, USA
Ras inhibitor (FTA)	Cayman Chemical, Ann Arbor, MI, USA
INSR inhibitor (Sc221730)<br>IGF-1R inhibitor (T9576)<br>PLC inhibitor (U73343)<br>Raf inhibitor (AZ628)	Santa Cruz Biotechnology, Dallas, TX, USA
TrkB inhibitor (ANA-12)	Selleck, Shanghai, China
IGF-1R antibody<br>phospho-IGF-1R antibody<br>PI3K antibody<br>phospho-PI3K antibody<br>Akt antibody<br>phospho-Akt antibody<br>phospho-TrkA antibody<br>PLC antibody<br>phospho-PLC antibody<br>PKC antibody<br>phospho-PKC antibody	Cell Signaling Technology, Boston, MA, USA
TrkA antibody	Beijing Biosynthesis Biotechnology Co., Ltd., Beijing, China
β-actin antibody<br>Goat anti-rabbit IgG secondary antibody<br>Goat anti-mouse IgG secondary antibody	Beijing CoWin Biotech Company, Beijing, China

Supplementary Figures

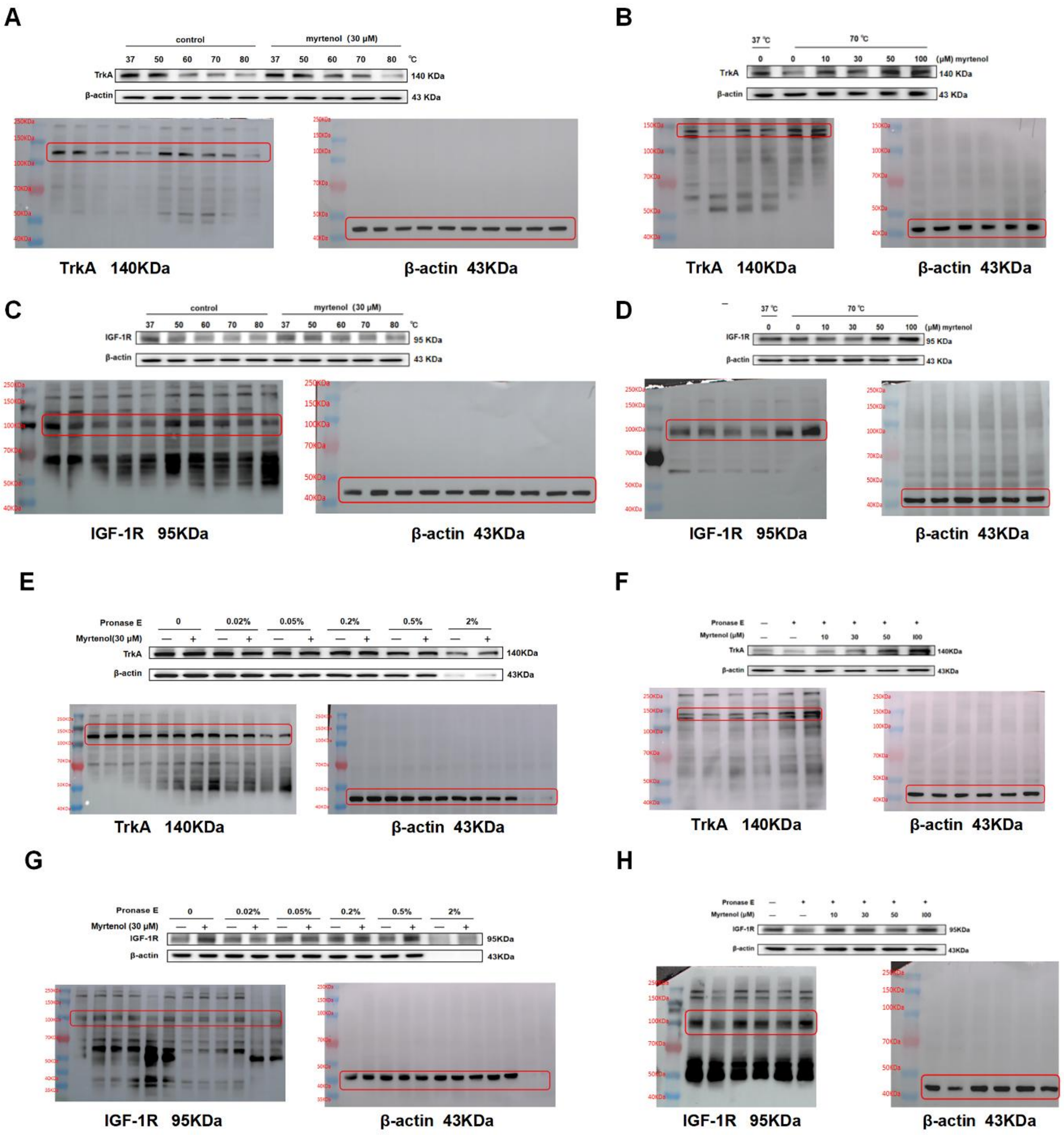

Supplementary Figure S1. Original data of Western blot analysis for identification of TrkA and IGF-1R as the target of myrtenol in Figure 4 and Supplementary Figure S1. (A) Original data of Western blot analysis of TrkA and β-actin proteins after being treated with myrtenol at 30 μM and heating the samples at different temperatures, respectively, in Figure 4A. (B) Original data of Western blot analysis of the binding stability between myrtenol and TrkA at varying compound concentrations in Figure 4B. (C) Original data of Western blot analysis of IGF-1R and β-actin proteins after being treated with myrtenol at 30 μM and heating the samples at different temperatures, respectively, in Figure 4C. (D) Original data of Western blot analysis of the binding stability between myrtenol and IGF-1R at varying compound

concentrations in Figure 4D. (E) Original data of Western blot analysis of TrkA and *β*-actin proteins after being treated with myrtenol at 30 μM and digested with different concentrations of pronase E, respectively, in Supplementary Figure S2. (F) Original data of Western blot analysis of myrtenol and *β*-actin proteins after being treated with different concentrations of myrtenol and digested with pronase E (0.5%) in Figure 4E. (G) Original data of Western blot analysis of IGF-1R and *β*-actin proteins after being treated with myrtenol at 30 μM and digested with different concentrations of pronase E, respectively, in Supplementary Figure S2. (H) Original data of Western blot analysis of myrtenol and *β*-actin proteins after being treated with different concentrations of myrtenol and digested with pronase E (0.5%) in Figure 4F.

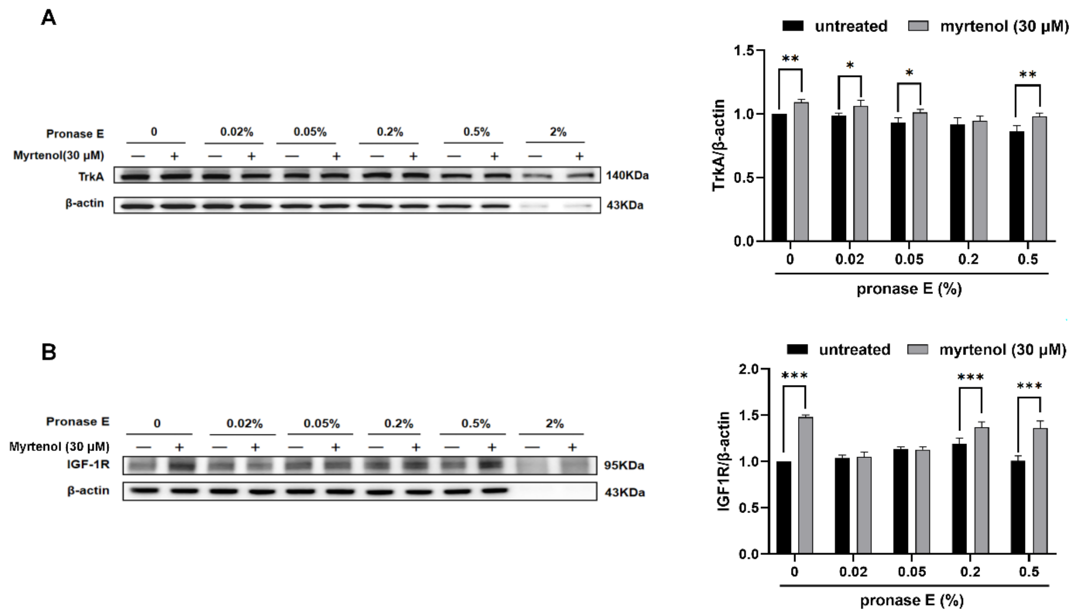

Supplementary Figure S2. Explore the optimal enzyme concentration for degradation of the target protein. Resistance to degradation by different concentrations of pronase E in PC12 cells treated without or with 30 μM myrtenol. (A) Western blot analysis and digital results of TrkA protein levels after adding different concentrations of pronase E. (B) Western blot analysis and digital results of IGF-1R protein levels after adding different concentrations of pronase E. Data are presented as mean ± SEM. $^{**}p < 0.01$, $^{***}p < 0.001$ indicated significant differences from the negative control groups.

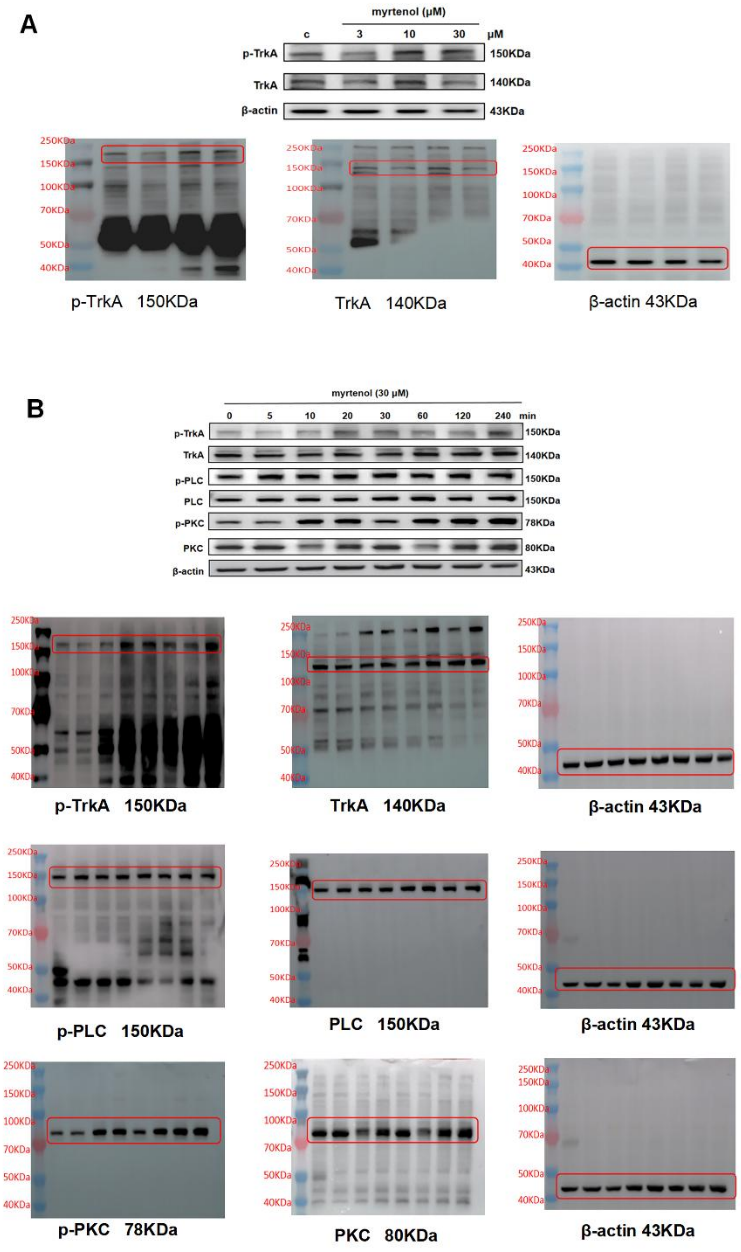

Supplementary Figure S3. Original data of Western blot analysis for investigation of the dose and time relationship of TrkA, PLC, and PKC phosphorylated induced by myrtenol in Figure 5A-5B. (A) Original data in Figure 5A represented the phosphorylation levels of TrkA induced by myrtenol in a dose-dependent manner. (B) Original data in Figure 5B represented the phosphorylation levels of TrkA, PLC, and PKC induced by myrtenol in a time-dependent manner.

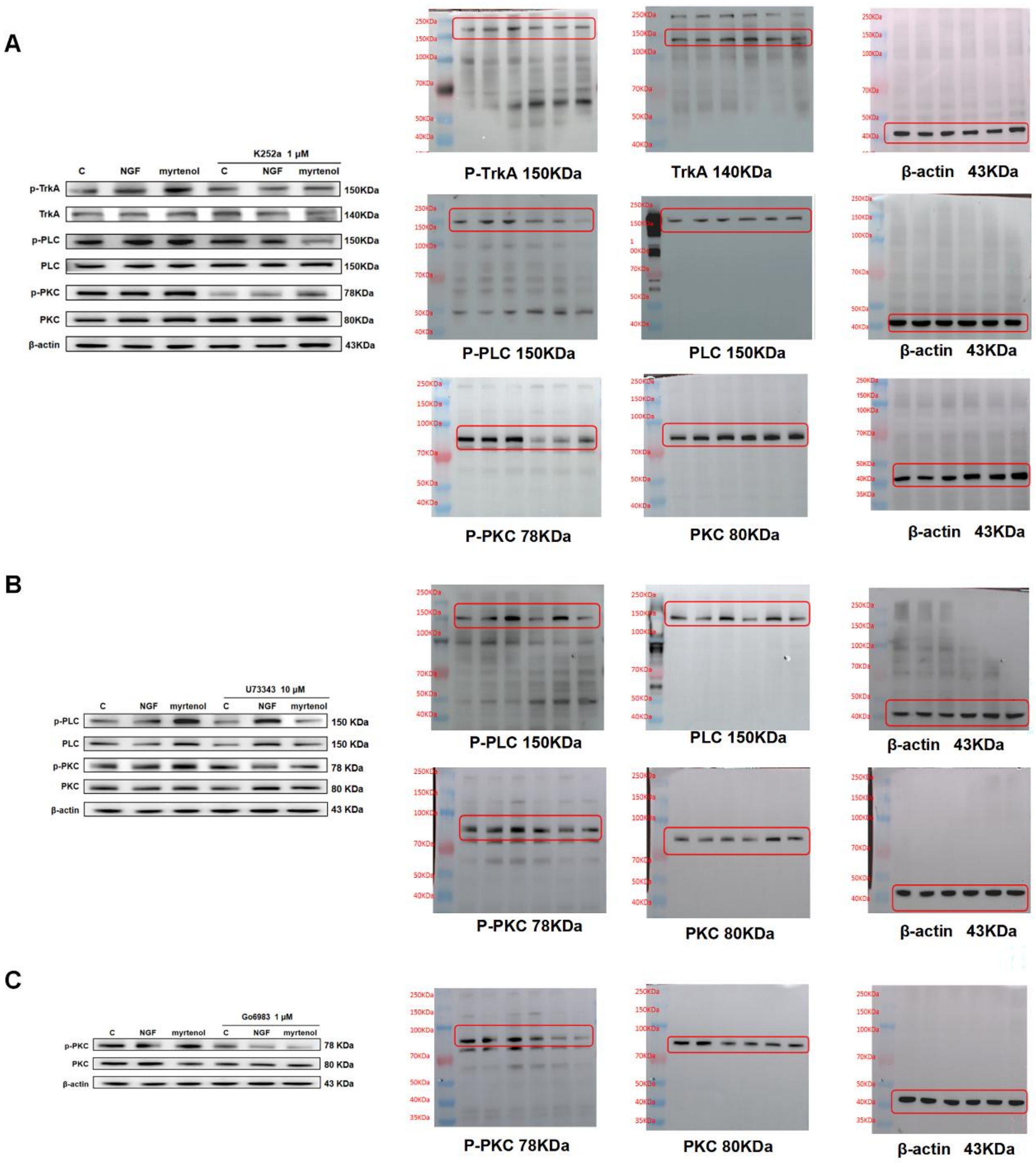

Supplementary Figure S4. Original data of Western blot analysis for determination of the relationship of TrkA, PLC, and PKC induced by myrtenol in Figure 6A-6C. (A) Original data of Western blot analysis of p-TrkA, TrkA, p-PLC, PLC, p-PKC, PKC and *β*-actin proteins treated with TrkA inhibitor and subsequently myrtenol or NGF treatment in Figure 6A. (B) Original data of Western blot analysis of p-PLC, PLC, p-PKC, PKC, and *β*-actin proteins after the treatment of PLC inhibitor and subsequently myrtenol or NGF treatment in Figure 6B. (C) Original data of Western blot analysis of p-PKC, PKC, and *β*-actin proteins after the treatment of PKC inhibitor and subsequently myrtenol or NGF treatment in Figure 6C.

A

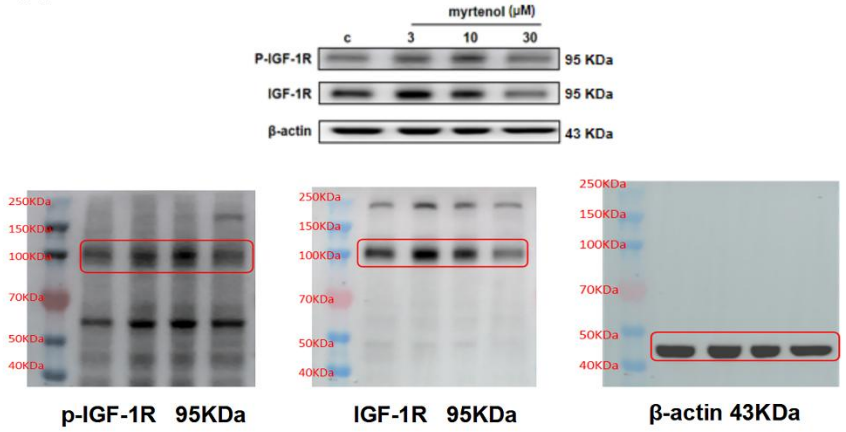

myrtenol (μM)
c
3
10
30
P-IGF-1R
95 KDa
IGF-1R
95 KDa
β-actin
43 KDa
250KDa
150KDa
100KDa
70KDa
50KDa
40KDa
p-IGF-1R 95KDa
IGF-1R 95KDa
β-actin 43KDa

B

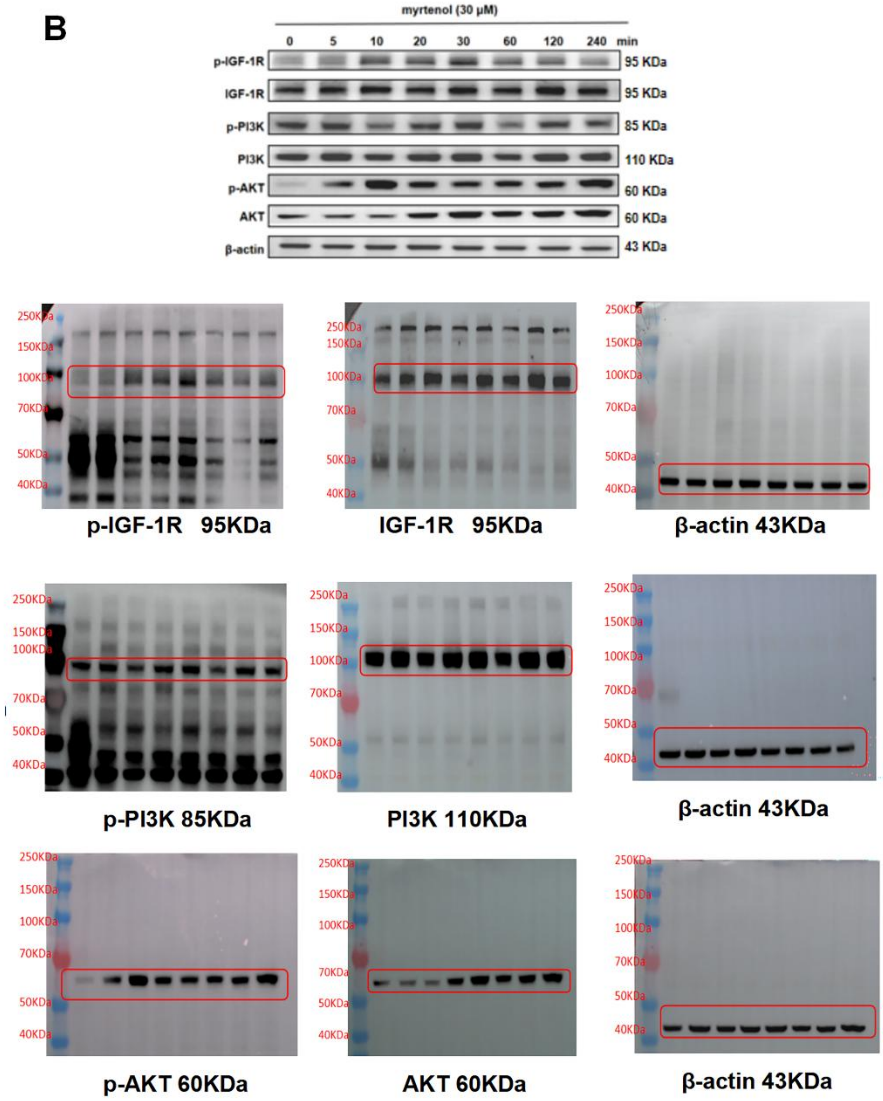

myrtenol (30 μM)
0
5
10
20
30
60
120
240
min
p-IGF-1R
95 KDa
IGF-1R
95 KDa
p-PI3K
85 KDa
PI3K
110 KDa
p-AKT
60 KDa
AKT
60 KDa
β-actin
43 KDa
250KDa
150KDa
100KDa
70KDa
50KDa
40KDa
p-IGF-1R 95KDa
IGF-1R 95KDa
β-actin 43KDa
p-PI3K 85KDa
PI3K 110KDa
β-actin 43KDa
p-AKT 60KDa
AKT 60KDa
β-actin 43KDa

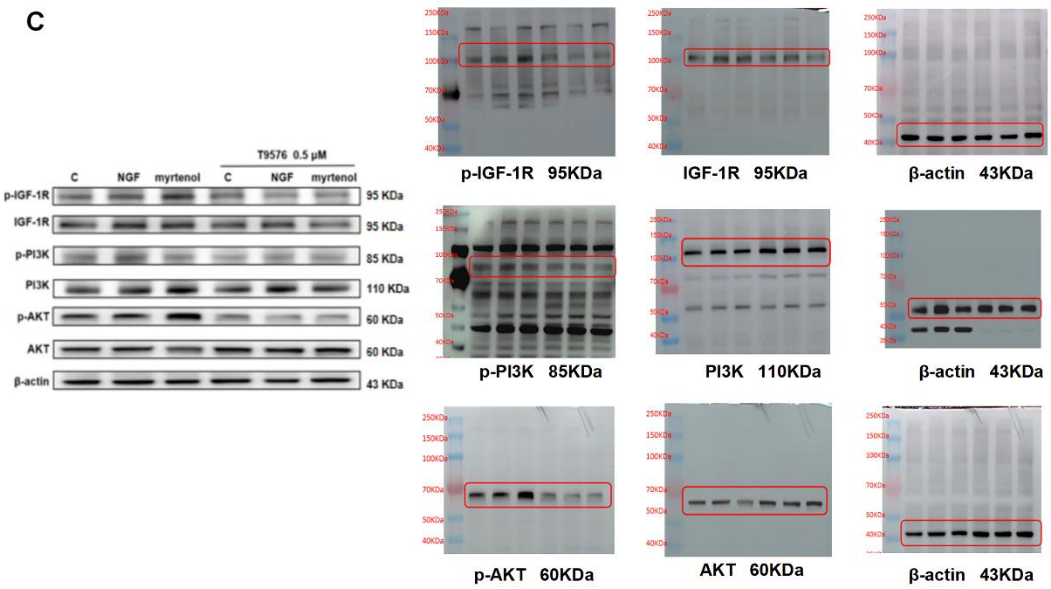

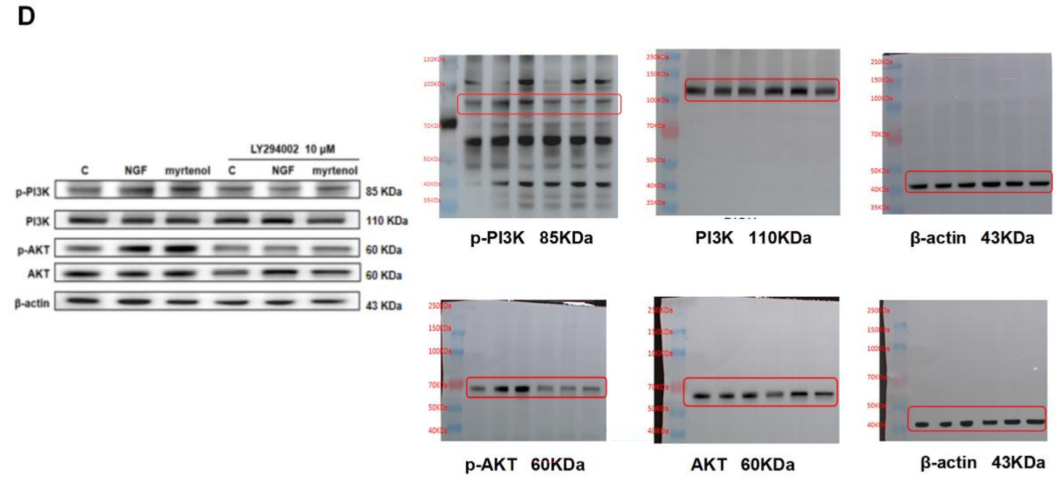

Supplementary Figure S5. Original data of Western blot analysis in Figure 7. (A) Original data in Figure 7A represented the phosphorylation levels of IGF-1R induced by myrtenol in a dose-dependent manner. (B) Original data in Figure 7B represented the phosphorylation levels of IGF-1R, PI3K, and AKT induced by myrtenol in a time-dependent manner. Original data of Western blot analysis for determination of the relationship of IGF-1R, PI3K, and AKT induced by myrtenol in Figure 7C-7D. (C) Original data of Western blot analysis of p-IGF-1R, IGF-1R, p-PI3K, PI3K, p-AKT, AKT and *β*-actin proteins treated with IGF-1R inhibitor and subsequently myrtenol or NGF treatment in Figure 7C. (D) Original data of Western blot analysis of p-PI3K, PI3K, p-AKT, AKT, and *β*-actin proteins after the treatment of PI3K inhibitor and subsequently myrtenol or NGF treatment in Figure 7D.

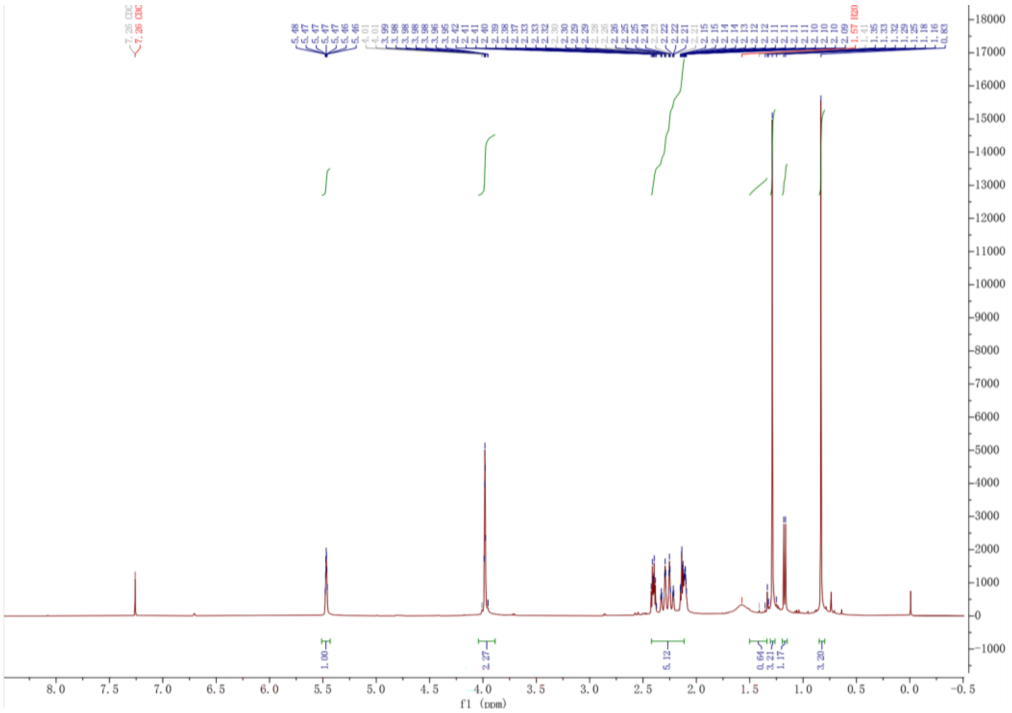

Supplementary Figure S6. The ^{1}H NMR spectrum of myrtenol.
